# AutoGERN: single-cell RNA-seq gene regulatory network inference via explicit link modeling and adaptive architectures

**DOI:** 10.1093/bioinformatics/btag143

**Published:** 2026-03-24

**Authors:** Jiacheng Wang, Yaojia Chen, Quan Zou, Ximei Luo

**Affiliations:** Institute of Fundamental and Frontier Sciences, University of Electronic Science and Technology of China, Chengdu, 611731, China; Yangtze Delta Region Institute (Quzhou), University of Electronic Science and Technology of China, Quzhou, Zhejiang, 324003, China; Institute of Fundamental and Frontier Sciences, University of Electronic Science and Technology of China, Chengdu, 611731, China; Yangtze Delta Region Institute (Quzhou), University of Electronic Science and Technology of China, Quzhou, Zhejiang, 324003, China; Institute of Fundamental and Frontier Sciences, University of Electronic Science and Technology of China, Chengdu, 611731, China; Yangtze Delta Region Institute (Quzhou), University of Electronic Science and Technology of China, Quzhou, Zhejiang, 324003, China; Institute of Fundamental and Frontier Sciences, University of Electronic Science and Technology of China, Chengdu, 611731, China; Yangtze Delta Region Institute (Quzhou), University of Electronic Science and Technology of China, Quzhou, Zhejiang, 324003, China

## Abstract

**Motivation:**

Single-cell RNA sequencing (scRNA-seq) enables transcriptome-wide profiling at single-cell resolution, revealing heterogeneous regulatory programs and making gene regulatory network (GRN) inference both central and challenging. Recent graph neural network (GNN)-based approaches for GRN inference typically model edges only implicitly (for example, via concatenated node embeddings), which limits their ability to capture complex regulatory dependencies. In addition, distributional shifts across scRNA-seq datasets make a single fixed GNN architecture poorly suited for broad generalization.

**Results:**

We present AutoGERN, a GNN framework tailored for GRN inference from scRNA-seq data. AutoGERN explicitly models regulatory information in the message-passing space and learns expressive link (edge) embeddings, which a lightweight multilayer perceptron uses to score gene–gene regulatory associations. To enhance flexibility and representational power, AutoGERN employs dual message-passing spaces (within-layer and cross-layer) and integrates a robust AutoGNN-based architecture search to adapt the network design to differing dataset distributions. Extensive experiments on multiple real scRNA-seq datasets demonstrate that AutoGERN consistently achieves superior performance and robustness compared with state-of-the-art baselines.

**Availability and implementation:**

The code and data of AutoGERN are available on GitHub at https://github.com/JChander/AutoGERN and on Zenodo at https://doi.org/10.5281/zenodo.18659807.

## Introduction

Single-cell RNA sequencing (scRNA-seq) provides high-resolution, transcriptome-wide measurements at the level of individual cells (Deng *et al.* 2014, Trapnell *et al.* 2014, Hou *et al.* 2016, Cao *et al.* 2021, Cao *et al.* 2022), transforming our understanding of cellular heterogeneity, lineage dynamics, and processes (Trapnell 2015, Wen and Tang 2016, Wang *et al.* 2026). Central to these is the inference of gene regulatory networks (GRNs) that govern differentiation and orchestrate state transitions (Basith *et al.* 2021, Phan *et al.* 2023), a problem that remains methodologically challenging (Luecken and Theis 2019, Wendt *et al.* 2020).

Early GRN inference methods based on unsupervised learning exploit only expression profiles, using correlation (Kim 2015, Specht and Li 2017) or mutual information (Chan *et al.* 2017, Qiu *et al.* 2020) for co-expression analysis, regression models guided by pseudo-time (Papili Gao *et al.* 2018, Sanchez-Castillo *et al.* 2018, Moerman *et al.* 2019), or causal reconstruction frameworks (Bonnaffoux *et al.* 2019, Deshpande *et al.* 2022). While these approaches can be effective when auxiliary information is scarce, their performance on real single-cell datasets is often limited and, in some cases, approaches randomness. Supervised strategies (Yuan and Bar-Joseph 2019, Chen *et al.* 2021, Fan and Ma 2021, Shu *et al.* 2021, Wang *et al.* 2023, Wang *et al.* 2025) address this by incorporating prior knowledge (e.g. known regulatory edges) during training, typically improving accuracy. However, most supervised pipelines operate at the level of isolated gene pairs and under-utilize the local topological structure of the regulatory graph—structure that is crucial for accurate GRN reconstruction.

Graph neural networks (GNNs) (Tran *et al.* 2025, Wang *et al.* 2025) have shown strong performance on single-cell analysis, including regulatory inference. Recent methods adapt GNNs to GRN inference in different ways: GRGNN (Wang *et al.* 2020) reframes GRN inference as subgraph classification around candidate gene pairs, GCNG (Yuan and Bar-Joseph 2020) integrates spatial context via cell–cell graphs, and GeneSpider (Du *et al.* 2023) together with GENELink (Chen and Liu 2022) transform expression and prior regulatory data into GNN-friendly representations for TF–target prediction and latent causal links. Despite these advances, most models still rely on fixed, manually designed GNN architectures whose performance often fails to transfer across heterogeneous scRNA-seq distributions, and they emphasize node embeddings while handling edges only implicitly, lacking explicit link representations needed to model complex regulatory dependencies such as directionality and causality.

To address these challenges, we introduce AutoGERN, a GNN framework tailored for GRN inference from scRNA-seq data. AutoGERN explicitly models regulatory information in the message-passing space by learning expressive link embeddings, which are subsequently scored by a lightweight multilayer perceptron to infer TF–target interactions. To increase representational flexibility, AutoGERN integrates two complementary message-passing spaces—intra-layer and inter-layer—capturing regulatory dependencies at multiple levels. In addition, AutoGERN employs a robust, AutoGNN-based architecture search procedure that adapts the GNN design to the distributional characteristics of each dataset, mitigating the brittleness of one-size-fits-all architectures.

We evaluate AutoGERN within the BEELINE (Pratapa *et al.* 2020) benchmarking protocol across seven canonical scRNA-seq datasets. Extensive experiments demonstrate that AutoGERN consistently outperforms state-of-the-art baselines in both accuracy and robustness. We further show that incorporating prior regulatory knowledge effectively guides training and yields additional gains, and that AutoGERN maintains strong performance under multiple negative-class imbalance settings commonly encountered in real-world scRNA-seq GRN inference.

## Methods

**Figure 1:**
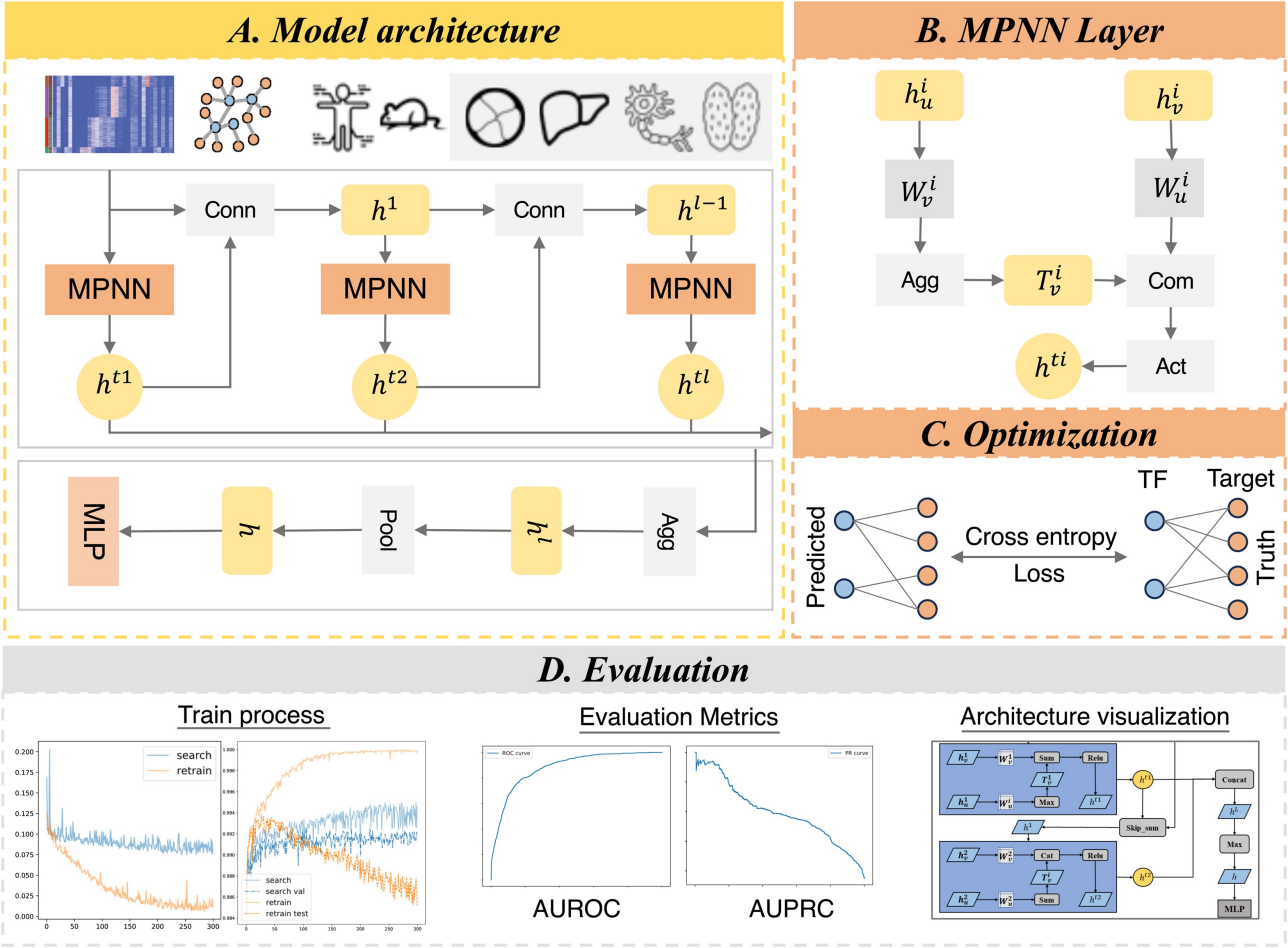
An overview of AutoGERN architecture. (A) The overall model architecture of AutoGERN. Single-cell RNA-seq dataset and network are curated from four tissues of human and mouse. (B) The structure of a message passing neural network layer. (C) We use cross-entropy to calculate the difference between the predicted GRNs and the ground truth as the loss to optimize the model parameters. (D) The trends of model loss and AUROC value during the architecture adaptation phase and the retraining phase, and the model’s adaptive architecture were recorded.

AutoGERN takes as input the scRNA-seq expression matrix X and the prior graph G. For each scRNA-seq dataset, we focus on a single annotated cell type and construct a gene prior graph at the cell-type level. The input expression matrix consists of all single cells assigned to that cell type, which are treated as node features in the constructed prior graph. AutoGERN explicitly learns link representations within the message-passing space to leverage and organize complex regulatory information. To enhance expressivity and flexibility, AutoGERN alternates link updates across two complementary message-passing spaces, an intra-layer space that strengthens within-layer relational cues and an inter-layer space that propagates cross-layer dependencies. After multiple rounds of aggregation, the resulting link embeddings are scored by a lightweight multilayer perceptron to infer TF–target interactions. AutoGERN learns a single GRN for each dataset/cell type, which we interpret as a cell-type–specific regulatory network. In addition, we incorporate a robust AutoGNN-based architecture search to adapt the GNN design to distributional differences across scRNA-seq datasets. The overall architecture of AutoGERN is summarized in Fig. 1.

### Message passing mechanism

AutoGERN adopts an intra-layer message passing framework that explicitly models edges between genes, allowing the network to learn dedicated representations of gene–gene regulatory associations rather than treating them only implicitly through node embeddings.


$$\begin{array}{c} T_{v}^{l+1}={\mathrm{Agg}}_{l}(\left\{W_{\delta (u)}^{l}h_{u}^{l},u\in N(v)\right\})\\ h_{v}^{l+1}=f_{l}({\mathrm{Com}}_{l}(\left\{W_{\mathrm{self}}^{l}h_{v}^{l},T_{v}^{l+1}\right\})) \end{array}$$

where *N*(*v*) represents the neighborhood set of node *v*, and $T_{v}$ is the intermediate embeddings collected from the neighborhood, *Agg* is the neighborhood aggregation method, $h_{v}$ and $h_{u}$ represent the latent embeddings of nodes *v* and *u* respectively. While *f* is the activation function, and *Com* is the combination function between the intermediate embeddings and the embedding of the previous layer node *v* itself. $W_{\delta (u)}^{l}$ is a domain-specific weight matrix encoding the link information in the graph, where δ(u) belongs to the set {self, neighbor}. This is a weak attention mechanism targeting basic link information in a graph, which can distinguish edges of its own type from those of the domain type.

We introduce residual and dense-style connections to stabilize optimization, promote feature reuse, and enable deeper GNNs. We formalize these cross-layer pathways as an inter-layer information passing mechanism and define a corresponding search space over layer connectivity and aggregation. Within this space, AutoGERN uses adaptive architecture search to select among stacked, skip-summation, and skip-concatenation schemes, yielding dataset-specific inter-layer configurations that generalize more reliably across heterogeneous scRNA-seq settings.


hl+1=Conn(hl,ht(l+1))={ht(l+1),stacksum(hl,ht(l+1)),skipsumWcnt(hl,ht(l+1)),skipconcat

where $h^{l}$ is the link embedding from the output of the message passing neural layer at layer *l*, which is then used as the input to the message passing layer at layer *l* + 1. This is then connected to the intermediate output $h^{t(l+1)}$ of the message passing layer at layer *l* + 1 and input into the next layer, iterating in this way. Beyond connectivity, the inter-layer aggregation module performs adaptive representation learning over multiple layers. Analogously, we define three candidate aggregation modes in this module: skip aggregation, concatenation aggregation, and max aggregation. By jointly searching over intra-layer and inter-layer operations, AutoGERN automatically discovers message-passing architectures that are well aligned with the underlying regulatory structures of different single-cell datasets.


hL=Agg(ht1,ht2,…,htl)={htl,skip[ht1||ht2||…||htl],concatmax⁡(ht1,ht2,…,htl),max

In the formula, the Agg layer aggregates the intermediate representations $h^{tl}$ learned from all the information passing layers and aggregates them into the final link embedding $h^{L}$ learned by the L-layer information passing layer.

### Pooling layer

After alternating between intra-layer and inter-layer information transfer, our model applies a pooling (readout) operation to obtain a higher-level representation of each link embedding $h^{L}$. For the link prediction task on the prior regulatory graph, this pooling step produces the final link representation between pairs of gene nodes.


$$h=\Gamma (\{h_{e}^{L}|e\in E\})$$

where Γ(·) represents the pooling operation, and *E* represents the set of all target links in the graph. In many AutoGNN-style models, subgraph-level pooling aggregates signals from the entire k-hop enclosing subgraph (Zhang and Chen 2018), whereas pairwise link prediction mainly requires a joint representation of the two endpoint nodes (Srinivasan and Ribeiro 2020). Motivated by DE-GNN (Li *et al.* 2020), we therefore adopt an endpoint-focused readout using simple sum, max, or concatenation pooling over the target node pair, which simplifies computation while better aligning the pooling mechanism with link-level prediction.

### Automatic search algorithm

AutoGERN performs automatic architecture search over a modular operation space $\Omega =\Omega_{\text{intra}}\cup \Omega_{\text{inter}}\cup \Omega_{\text{pool}}$. The intra-layer space $\Omega_{\text{intra}}$ covers candidate weight matrices, neighborhood aggregation functions (mean, sum, max), feature combination schemes (additive/residual, concatenation) and activation functions. The inter-layer space $\Omega_{\text{inter}}$ controls cross-layer information flow via stacked, skip-summation and skip-concatenation connections and aggregation rules. And the pooling space $\Omega_{\text{pool}}$ defines link-level readout operators (sum, max, concatenation).

For the hidden feature vector $h^{l}$ that needs to be connected, assuming the weight of the chosen connection operation ${\mathrm{Conn}}_{l}$ is $a_{\mathrm{conn}}$, then the output of this inter-layer connection function can be expressed by formula:


$$\bar{{\mathrm{Conn}}_{l}}(h^{l})=\sum_{{\mathrm{Conn}}_{l}\in ο}I_{{\mathrm{Conn}}_{l}}\cdot {\mathrm{Conn}}_{l}(h^{l})$$

where ${\mathrm{IConn}}_{l}\in \{0,1\}$, and when one candidate operation is 1, the other two candidate operations are 0. Assuming that θ is the set of operations selected from all candidate operations in our model, then the search task of the graph neural network model can be formalized as shown in formula:


$$\max_{\theta ,\omega }E\mathrm{val}(\theta ,\omega ;G)$$

where *Eval*(·) is the predictive performance of the model operation combination θ with weight ω on the prior control graph G.

We adopt a stochastic yet differentiable architecture search scheme that treats the operator on each edge as a categorical random variable and uses Gumbel–Softmax-style reparameterization to draw approximately one-hot samples from a learned distribution ρβ(θ). During training, the network effectively runs in discrete mode, activating a single operator per edge in each forward pass, so that optimization and inference regimes are aligned, the performance gap between searched and final architectures is reduced, and computation is lowered while retaining gradient-based learning of the architecture parameters.


$$\theta_{o}=\frac{\exp((\log\beta_{o}-\log(-\log(U_{o})))/\tau )}{\sum_{o^{\prime }\in O}\exp((\log\beta_{o^{\prime }}-\log(-\log(U_{o^{\prime }})))/\tau )}$$

where o is a certain operation among the candidate operations, $U_{o}∼\mathrm{Uniform}(0,1)$ is uniformly distributed sampling, and τ represents the tolerance value of the softmax activation function. This firstly ensures that the probability of o being sampled (i.e. $\theta_{o}=1$) is proportional to its weight $\beta_{o}$. At the same time, the one-hot property $\lim_{\tau \to 0}\theta_{o}=1$ makes the random differentiable relaxation unbiased after convergence. Therefore, the adaptive problem of the graph neural network model is reformulated into formula, where E[⋅] is expect function:


$$\max_{\beta ,\omega }E_{\theta ∼\rho \beta (\theta )}[\mathrm{Eval}(\theta ,\omega ;G)]$$

### Model implementation

We implemented AutoGERN building upon on the AutoGEL framework (Wang *et al.* 2021). In all experiments, we used a two-layer message-passing backbone, with each layer producing a 256-dimensional hidden representation. Model parameters were optimized using the Adam optimizer with a learning rate of 1 × 10^−4^. To mitigate overfitting, we applied Dropout with a rate selected from $\left\{0,0.2\right\}$. The mini-batch size was set to 128, the total number of training epochs to 20, and the number of adaptive architecture search (iteration) epochs to 20. More details of model implementation can be seen in the Supplementary Notes.

### Datasets

To assess GRN inference during real cellular development, we assembled seven scRNA-seq benchmark datasets from BEELINE (Pratapa *et al.* 2020), spanning two human cell types, hESC (human embryonic stem cells) (Chu *et al.* 2016) and hHEP (human mature hepatocytes) (Camp *et al.* 2017), and five mouse cell types, mESC (mouse embryonic stem cells) (Shimosato *et al.* 2007), mDC (mouse dendritic cells) (Shalek *et al.* 2014), and mHSC (mouse hematopoietic stem cells) (Nestorowa *et al.* 2016) with three lineages (mHSC-E erythroid, mHSC-L lymphocyte, and mHSC-GM granulocyte–macrophage). For each cell type, ChIP-seq data were obtained from ChIP-Atlas (Oki *et al.* 2018) to construct cell-type-specific GRNs, which serve as the reference networks for benchmarking. ChIP-seq-based GRNs provide mechanistically grounded and direction-aware supervision, which is preferable to purely correlation-based gold standards for supervised GRN inference. It is also important to recognize that such reference networks are at best a silver standard rather than a complete and error-free representation of the in vivo GRN. More discussions on ChIP-seq data can be seen in Supplementary Notes.

### Preprocessing

All seven scRNA-seq datasets were processed with Scanpy (Wolf *et al.* 2018). Following the BEELINE configuration for each dataset, we first removed low-quality cells and filtered out genes expressed in fewer than 90% of cells. Library-size normalization was then performed (TPM/CPM), followed by log transformation of the normalized counts. In line with BEELINE, we identified highly variable genes using a Bonferroni-corrected significance threshold of α = 0.01, and retained the top 500 and top 1000 most variable genes for downstream benchmarking. Transcription factors (TFs) were curated from TRRUST (Han *et al.* 2015) and RegNetwork (Liu *et al.* 2015). After deduplication, all curated TFs were included in each dataset. Finally, we constructed the feature panels in a cell-type-specific manner by combining the highly variable TFs with the top 500 or top 1000 non-TF genes.

### Evaluation strategy

For each benchmark dataset, we adopt a hold-out strategy to construct edge-disjoint training, validation and test partitions of the gold-standard regulatory edges in an 80%, 10%, and 10% ratio (More details of dataset splitting and negative sampling in Supplementary notes, and Supplementary Tabel S3). For evaluation metrics, performance is assessed with the Area Under the ROC Curve (AUROC) and the Area Under the Precision–Recall Curve (AUPRC). Both range from 0 to 1, with larger values indicating better performance.

## Results

### AutoGERN outperforms existing methods in cell-type-specific GRNs inference

**Figure 2:**
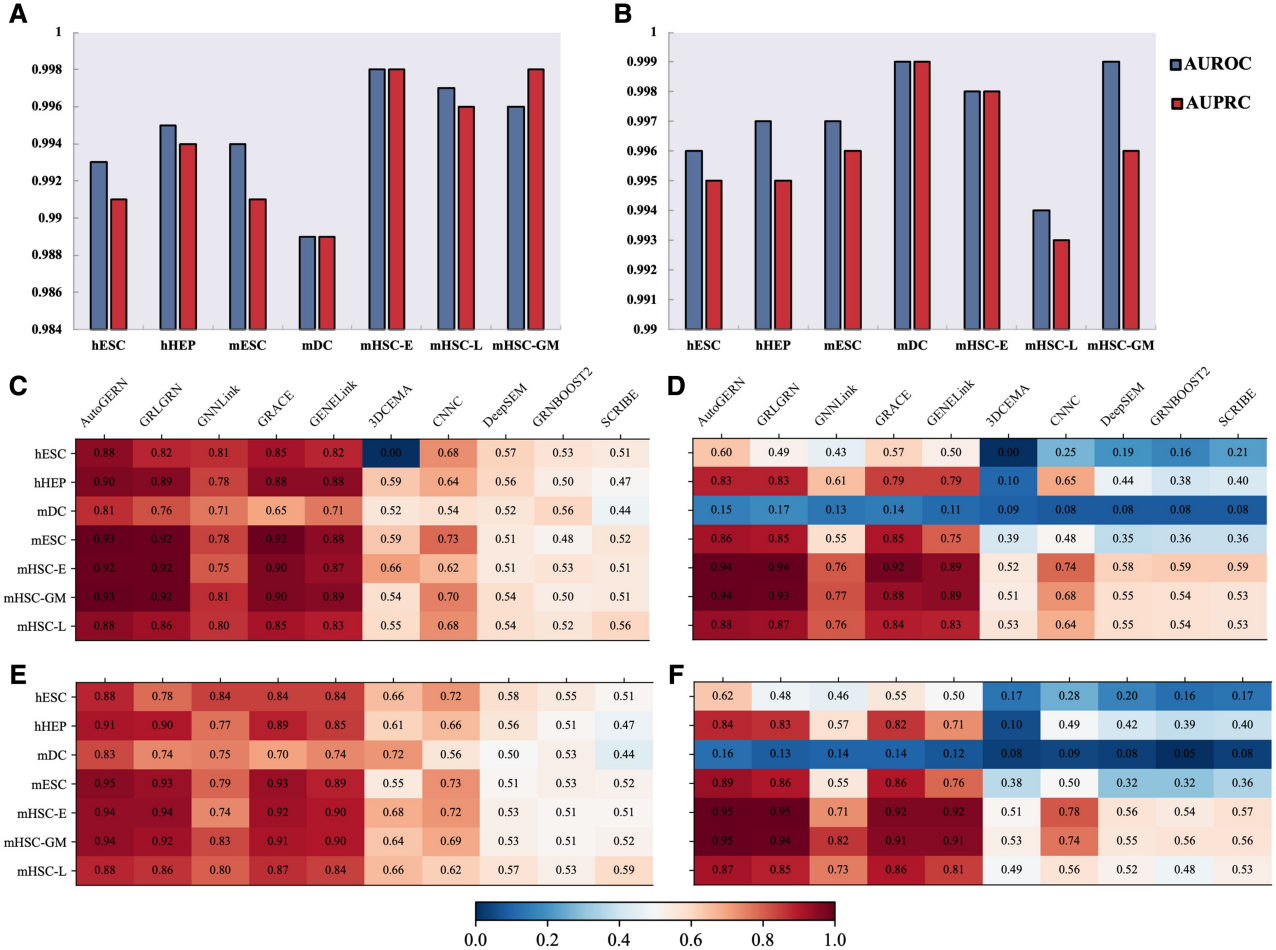
Inference performance of AutoGERN and other state-of-the-art models on seven scRNA-seq benchmark datasets in terms of AUROC and AUPRC values. The performance of AutoGERN on benchmark datasets with 500 genes (A) and 1000 genes (B). (C-F) The performance on benchmark datasets in the cold-start scenario, where train-valid-test set was split by using “Hard Split” strategy to avoid the risk of data leakage. For benchmark datasets with 500 most significantly varying genes, we compared AutoGERN against other SOTA methods in terms of AUROC (C) and AUPRC (D) values. For datasets with 1000 most significantly varying genes, models were also benchmarked in terms of AUROC (E) and AUPRC (F) values.

We applied it to seven real scRNA-seq data and assessed GRN inference performance. AutoGERN achieves excellent performance on all seven 500-gene datasets, with AUROC and AUPRC both exceeding 0.98 (Fig. 2A). Performance is highest on the three mHSC datasets (both metrics >0.99) and slightly lower on mDC, likely reflecting differences in the density and completeness of prior regulatory knowledge. When the gene set is expanded to 1000 genes, AutoGERN further improves on most datasets and attains AUROC and AUPRC values above 0.99 across all seven benchmarks, with particularly pronounced gains on mDC.

Under the Hard Split strategy, we benchmarked AutoGERN on these scRNA-seq datasets against six supervised baselines: CNNC (Yuan and Bar-Joseph 2019), 3DCEMA (Fan and Ma 2021), GENELink (Chen and Liu 2022), GRACE (Wang *et al.* 2025), GNNLink (Mao *et al.* 2023), GRLGRN (Wang *et al.* 2025), and three unsupervised models [SCRIBE (Qiu *et al.* 2020), GRNBOOST2 (Moerman *et al.* 2019), DeepSEM (Shu *et al.* 2021)]. In the 500-gene setting, AutoGERN attains the best performance on 13 of 14 benchmark tasks in terms of both AUROC (Fig. 2C) and AUPRC (Fig. 2D), improving on the second-best method, GRLGRN, by an average of 2.3% AUROC and 1.7% AUPRC, with particularly large AUROC gains on hESC (6%) and mDC (5%) and an 11% AUPRC improvement on hESC.

**Figure 3:**
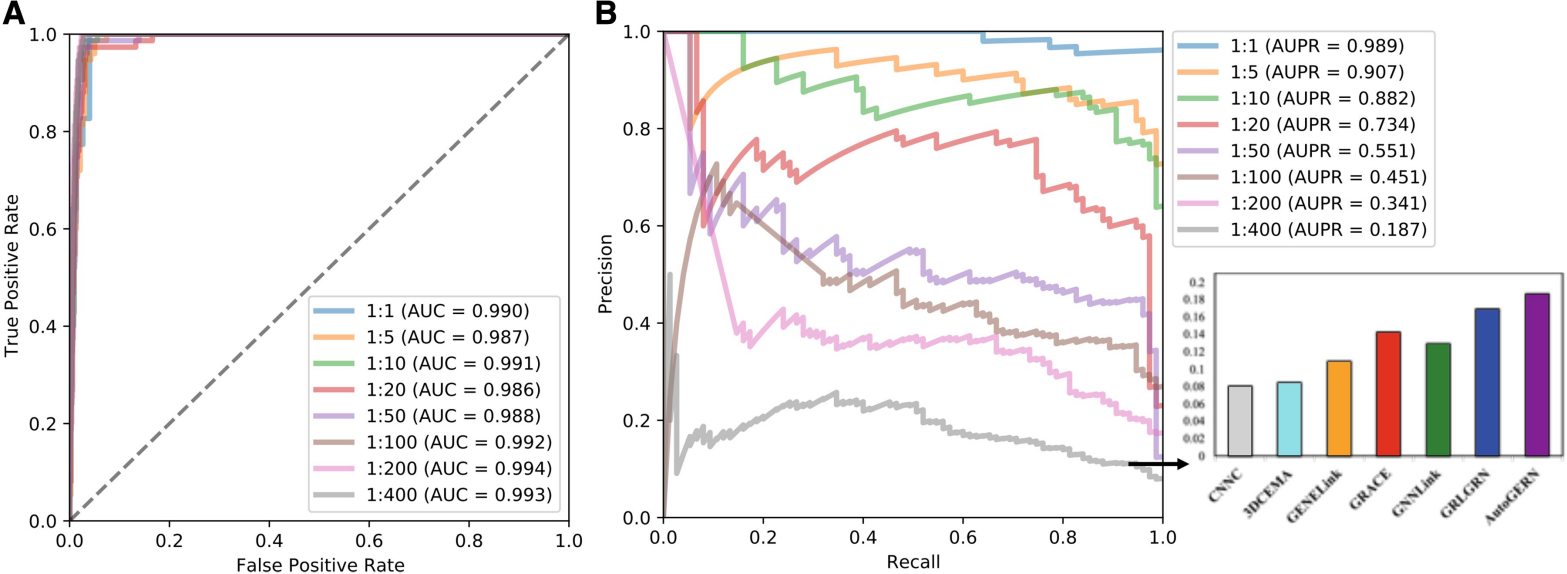
Performance evaluation of the AutoGERN model in various scenarios with imbalanced positive and negative samples. The model was benchmarked on the mDC dataset with positive and negative sample ratios of 1:1, 1:5, 1:10, 1:20, 1:50, 1:100, 1:200, and 1:400, and its performance in AUROC (A) and AUPRC (B) was evaluated. (B) Comparison of AutoGERN’s performance with six state-of-the-art supervised learning algorithms for gene regulation network inference in a scenario with a positive and negative sample ratio of 1:400.

In the 1000-gene setting, AutoGERN achieves the best AUROC and AUPRC on all 14 benchmark tasks (Fig. 2E, F), with average improvements of 3.9% and 3.4%, respectively, over the second-best model and a maximal AUROC gain of 14% on hESC. Notably, the performance gains on the 1000-gene datasets are nearly twice those in the 500-gene setting, supporting the view that richer prior regulatory knowledge enables AutoGERN to more effectively learn and generalize underlying GRN patterns from single-cell expression data, and highlighting its robustness when prior information is sparse or incomplete. Moreover, we provide the computational complexity and efficiency on 14 benchmark datasets (Fig. 3, available as supplementary data at *Bioinformatics* online). The discussion about scalability of AutoGERN can be found in Supplementary Notes.

### AutoGERN has good robustness on imbalanced positive-negative samples

Among seven real scRNA-seq datasets, the mDC GRN exhibits a positive-to-negative ratio of ∼1:400, which can bias models toward predicting most unknown relationships as non-regulatory, especially in the presence of noisy and imperfect biological labels.

During training, AutoGERN first uses standard negative sampling, randomly drawing an equal number of unlabeled TF–gene pairs as negatives to balance the classes. However, this setting underestimates the severity of imbalance in real systems. We therefore systematically evaluated AutoGERN on the mDC dataset under a series of increasingly imbalanced regimes, fixing the positive-to-negative ratio at 1:5, 1:10, 1:20, 1:50, 1:100, 1:200, and 1:400, and measuring AUROC and AUPRC.

As shown in Fig. 3A, AUROC remains high and stable (≈0.99, fluctuating within 0.004) across all imbalance levels, but AUROC can be misleading under extreme imbalance because a model may predict almost all edges as negative and still appear strong. We therefore focus on AUPRC, which better reflects performance on the minority positive class by emphasizing the trade-off between precision and recall. As the number of negatives increases, AutoGERN’s AUPRC gradually declines (Fig. 3B), yet under the most extreme setting (1:400) it still substantially outperforms six supervised GRN inference baselines, improving AUPRC by nearly 10% over the second-best method, GRLGRN. These results highlight the robustness of AutoGERN in highly imbalanced, realistic GRN inference scenarios.

### Ablation study

AutoGERN employs several mechanisms to mitigate gradient vanishing and over-smoothing in graph neural networks and to support an adaptive search process, including inter-layer adaptation, global pooling, and a stochastic differentiable architecture search algorithm. To disentangle the relative contributions of these components and assess the robustness of the overall framework, we conducted a series of ablation experiments. These ablations help identify which sub-modules are critical for the performance and functionality of the complex system, and how AutoGERN behaves under different architectural constraints. Here, we focus on the changes in AutoGERN’s performance on seven real scRNA-seq datasets (each restricted to the 500 most variable genes) when disabling the following two sub-modules:

1. To examine the specific contribution of the inter-layer search space in AutoGERN framework, we removed it and retained only the set of operations in the intra-layer search space, yielding the variant AutoGERN-w/o-inter.
2. AutoGERN provides three different candidate pooling operators: sum pooling, average pooling, and max pooling. To evaluate the impact of this candidate pooling set, we removed the adaptive choice and replaced it with a fixed differentiable pooling operator, resulting in the variant AutoGERN-w/o-diffP.

As reported in Table 1, AutoGERN-w/o-diffP exhibits performance drops of more than 0.4 percentage points on the hESC and mDC datasets, while AutoGERN-w/o-inter shows reductions of more than 0.2 percentage points on six of the seven datasets (with the exception of mHSC-L). These results indicate that the inter-layer search space generally plays a more important role than adaptive global pooling on most datasets. However, on a subset of single-cell datasets with lower average node degree—such as mDC—the model is more sensitive to the choice of global pooling, and the removal of adaptive pooling leads to a more pronounced degradation. At the same time, neither variant exhibits a substantial overall performance collapse on the seven datasets, highlighting the strong capacity provided by the intra-layer search space alone. Notably, both ablated versions of AutoGERN, despite lacking these two components, still outperform the six other supervised baselines on all seven scRNA-seq datasets, underscoring the robustness and effectiveness of the proposed framework.

**Table 1:** Ablation analysis of the AutoGERN model on seven scRNA-seq datasets with 500 most significantly varying genes. AutoGERN was benchmarked against six supervised methods.

Methods	hESC	hHEP	mDC	mESC	mHSC-E	mHSC-GM	mHSC-L
CNNC	0.68	0.64	0.54	0.73	0.62	0.7	0.68
3DCEMA	0	0.59	0.52	0.59	0.66	0.54	0.55
GENELink	0.82	0.88	0.71	0.88	0.87	0.89	0.83
GRACE	0.85	0.88	0.65	0.92	0.9	0.9	0.85
GNNLink	0.81	0.78	0.71	0.78	0.75	0.81	0.8
GRLGRN	0.82	0.89	0.76	0.92	0.92	0.92	0.86
AutoGERN-w/o-diffP	0.876	0.898	0.8	0.926	0.922	0.926	0.876
AutoGERN-w/o-inter	0.878	0.897	0.804	0.926	0.921	0.925	0.875
AutoGERN	0.88	0.899	0.81	0.928	0.923	0.927	0.876

### Case studies: visualization of the AutoGERN model’s adaptive architecture

Each scRNA-seq data exhibits a distinct expression distribution, dropout rate and noise level, which in turn induces shifts in GRN structure. We visualize these shifts (Fig. 1, available as supplementary data at *Bioinformatics* online) by comparing the average degree and edge density of the gold-standard GRNs for each dataset.

Rather than relying on a single fixed design, AutoGERN adaptively searches over a space of GNN architectures to identify a suitable model for each scRNA-seq dataset. Given the pronounced distribution shifts in expression and the resulting differences in GRN topology (Fig. 1, available as supplementary data at *Bioinformatics* online), we examined whether the search procedure truly adapts to these heterogeneous regimes by summarizing the operations selected on all seven datasets (Table 2).

**Table 2:** AutoGERN features an adaptive graph neural network architecture on seven single-cell datasets.

Datasets	Agg	Combine	Activation	Layer Connect	Layer Agg	Pool
hESC	Max, Max	Sum, Sum	Relu, Relu	Skip_sum, skip_sum	None	Max
hHEP	Max, Sum	Sum, Concat	Relu, Relu	Skip_sum, Skip_cat	Concat	Max
mDC	Sum, Sum	Sum, Sum	Prelu, Relu	Stack, Skip_cat	Max_pool	Max
mESC	Sum, Sum	Sum, Sum	Relu, Relu	Skip_cat, Skip_sum	Max_pool	Concat
mHSC-E	Max, Max	Sum, Sum	Prelu, Relu	Stack, Skip_cat	Max_pool	Concat
mHSC-L	Max, Sum	Concat, Sum	Relu, Prelu	Stack, Stack	Max_pool	Sum
mHSC-GM	Sum, Max	Sum, Sum	Relu, Prelu	Stack, Stack	None	Max

Overall, the discovered architectures are clearly distinct, indicating that the search does not collapse to a single “one-size-fits-all” solution. Within the intra-layer search space, summation is the predominant feature-merging operation, with concatenation appearing only in a few layers, suggesting that simple additive fusion usually suffices while concatenation is reserved for cases requiring more expressive local integration. In the inter-layer space, the choice among stacking, skip-summation and skip-concatenation depends on network density: denser GRNs tend to favor residual-style connections that strengthen cross-layer information flow, whereas sparser graphs more often select plain stacking. For multi-level and global aggregation, max pooling is the dominant strategy, with occasional use of concatenation, implying that the searched architectures preferentially emphasize the strongest regulatory signals when extracting node- and edge-level patterns from noisy single-cell data. An example of the searched architecture for the hHEP dataset is shown in Fig. 2, available as supplementary data at *Bioinformatics* online.

For inferred GRNs interpretabilities, we further visualized CTCF (a TF)-centered module of mDC + 500 genes dataset by selecting the TF hub with the highest out-degree among 0.5% top-scoring edges and extracting its top-50 predicted targets (Fig. 4, available as supplementary data at *Bioinformatics* online; Supplementary Notes). The target set shows significant enrichment for immune regulatory processes (e.g. cytokine-mediated signaling) and pathways related to immune differentiation and antigen-processing machinery (e.g. Th17 differentiation and proteasome; Table 2, available as supplementary data at *Bioinformatics* online, FDR < 0.05). Notably, 13 of the top-50 targets are supported by the ChIP-seq reference network, suggesting that AutoGERN’s high-confidence module captures biologically coherent and partially validated regulatory signals.

## Conclusion

We proposed AutoGERN, a GNN framework for GRN inference from single-cell gene expression that explicitly models gene–gene interactions in the message-passing space and learns link-level embeddings in complementary intra-layer and hierarchical spaces. AutoGERN further incorporates a stochastic differentiable architecture search procedure that automatically discovers dataset-specific GNN architectures adapted to the distributional characteristics of each scRNA-seq dataset. Extensive experiments on seven real scRNA-seq benchmarks show AutoGERN consistently outperforms SOTA GRN inference methods and substantially improves both training and predictive performance. The model remains robust under multiple imbalanced scenarios and learns architectures aligned with the heterogeneous distributional and topological properties of different single-cell datasets, highlighting its practical utility for GRN inference in realistic single-cell settings. Despite these strengths, AutoGERN has several limitations. First, its performance gains come at the cost of computational and memory overhead due to the explicit modeling of gene-gene interactions, which reduces its spatiotemporal efficiency. Second, the model’s reliance on prior regulatory knowledge leads to performance degradation when such information is sparse, as it receives weaker supervision for distinguishing true regulatory relationships. In addition, AutoGERN is configured to infer one GRN per dataset or cell type, using single-cell data primarily to obtain clean, cell-type–specific expression distributions (Jeon *et al.* 2022) and use ChIP-seq-derived TF-target regulations as supervision. As such, the inferred networks are restricted as steady-state, cell-type–level regulatory graphs rather than fully dynamic, cell-state–resolved GRNs. An important next step is to integrate AutoGERN with perturbation-based single-cell assays, such as CRISPR Perturb-seq. In such settings, perturbation readouts can provide functional labels for TF–target regulations or be modeled as condition-dependent inputs, enabling AutoGERN to learn causal, perturbation-aware regulatory graphs and to predict gene expression changes under novel perturbations.

## Supplementary Material

btag143_Supplementary_Data
