# Supplementary material for: AutoGERN: single-cell RNA-seq gene regulatory network inference via explicit link modeling and adaptive architectures
Source: Bioinformatics. 2026 Mar 24;42(4):btag143. doi: 10.1093/bioinformatics/btag143 (PMC13064981; doi:10.1093/bioinformatics/btag143)
Supplement: btag143_Supplementary_Data [file btag143_supplementary_data.docx]

**AutoGERN: Single-Cell RNA-Seq Gene Regulatory Network Inference via Explicit Link Modeling and Adaptive Architectures**

Jiacheng Wang, Yaojia Chen, Quan Zou, Ximei Luo

## **Supplementary** **Notes**

**Dataset split and negative sampling**

The validation set is used exclusively for early stopping and hyperparameter selection, while the test set remains untouched until final evaluation. Let $T_{i}$ denote a transcription factor and $G_{j}$ a gene. Positives are observed regulatory pairs $(T_{i},G_{j})$ from the gold standard. Negatives are defined as unobserved TF–gene pairs $(T_{i},G_{nt})$ that do not appear as positives in any split. For each split, we construct a balanced set by sampling one negative per positive (that is 1:1 ratio), without replacement and without cross-split overlap. Sampling is stratified by TF to preserve the marginal $T_{i}$ distribution across classes. Unless otherwise specified, a fixed random seed is used to ensure reproducibility.

To obtain a more stringent and realistic evaluation, we also adopted a “Hard Split” setting in which all remaining TF–gene pairs were treated as candidate negatives and proportionally partitioned into train, validation (10%), and test sets, thereby avoiding information leakage and forcing the model to discriminate true targets from a large pool of unobserved pairs.

In the main experiments, negative samples are drawn uniformly at random from all unlabeled TF–gene pairs to form a balanced 1:1 positive-to-negative training set. To assess the robustness of AutoGERN to this choice, we additionally evaluated a co-expression–filtered negative sampling strategy on the mDC 500-gene dataset. In this scheme, we first compute TF–target co-expression across single cells and remove TF–gene pairs with high absolute correlation (corr > 0.5) from the negative candidate pool, and then sample negatives uniformly from the remaining pairs. All other settings (architecture search, training protocol, and evaluation metrics) are kept identical to the default configuration. We apply this high co-expression filtering schema both under the standard setting and under the Hard Split setting to examine whether the performance of AutoGERN substantially depends on the precise negative sampling protocol.

On the standard mDC 500-gene setting, we found that replacing uniform random negatives with co-expression–filtered negatives led to almost unchanged performance. The AUROC and AUPRC values remained at essentially the same levels as in the default setting (see Table S3), with no systematic degradation. Moreover, when we applied this high co-expression filtering for candidate negatives to the Hard Split scenario on the same dataset, AUPRC slightly increased compared with uniform random negatives (see Table S3), indicating that AutoGERN can even benefit from a more conservative, co-expression–aware choice of negative edges in the most imbalanced and challenging regime.

**Ground truth**

In this study, we use ChIP-seq–derived gene regulatory networks (GRNs) as reference (“ground truth”) for supervised training and evaluation. While ChIP-seq provides mechanistically grounded and direction-aware evidence of transcription factor (TF) binding, it is important to recognize that such reference networks are at best a silver standard rather than a complete and error-free representation of the in vivo GRN. First, most ChIP-seq experiments are performed in bulk and often in cell lines or tissues that do not perfectly match the cell types, developmental stages, or experimental conditions of the scRNA-seq datasets analyzed here. As a result, context-dependent regulatory interactions may be either missing from, or spuriously present in, the reference network. Second, the absence of a TF–gene pair from the ChIP-seq–derived GRN does not guarantee the absence of a regulatory interaction. It may simply reflect limited coverage, insufficient sequencing depth, conservative peak-calling thresholds, or lack of ChIP data for a given TF or condition. Thus, our “negative” edges are more accurately interpreted as unlabeled candidate pairs, and the evaluation should be viewed as measuring agreement with ChIP-supported interactions rather than recovery of all true regulatory edges.

Third, even when TF binding is confidently detected, not all binding events are functionally regulatory, and the mapping from ChIP-seq peaks to target genes is itself imperfect, especially for distal enhancer–promoter interactions. These factors introduce both false positives and false negatives (functional regulation without detectable binding or without correct peak-to-gene assignment) into the reference network. Finally, available ChIP-seq resources cover only a subset of TFs and conditions, leading to an incomplete and biased view of the underlying GRN. Taken together, these limitations imply that our ChIP-seq–based GRNs provide a useful but partial supervisory signal. AutoGERN’s performance should therefore be interpreted as its ability to recover ChIP-supported regulatory edges, with the understanding that the true biological GRN is broader and more context-dependent than what is captured by the reference.

**Evaluation metrics**

For a link prediction task, it essentially constitutes a binary classification problem for each potential link between gene pairs. Therefore, specific statistical metrics are typically employed to evaluate the performance of such classification. Let TP, FP, TN, and FN denote the numbers of true positives, false positives, true negatives, and false negatives, respectively. For a given decision threshold τ, the true positive rate (TPR, or sensitivity/recall) and false positive rate (FPR) are defined as:

$$TPR(\tau)= \frac{TP(\tau)}{TP(\tau)+FN(\tau)}$$

$$FPR(\tau)= \frac{FP(\tau)}{FP(\tau)+TN(\tau)}$$

Varying $\tau$ from 0 to 1 produces a receiver operating characteristic (ROC) curve by plotting

$TPR(\tau)$ against $FPR(\tau)$. The Area Under the ROC Curve (AUROC) is defined as the area under this curve and is computed numerically, for example by a trapezoidal rule over the sorted thresholds. AUROC ranges from 0 to 1, with 0.5 corresponding to random ranking and larger values indicating better global discrimination between positive and negative edges.

For the same predictions, precision and recall at threshold τ are defined as:

$$Precision(\tau)= \frac{TP(\tau)}{TP(\tau)+FP(\tau)}$$

$$Recall(\tau)= \frac{TP(\tau)}{TP(\tau)+FN(\tau)}$$

By varying $\tau$, we obtain a precision–recall (PR) curve by plotting Precision(τ) against Recall(τ). The AUPRC is the area under this curve, again estimated by numerical integration over all thresholds. AUPRC also lies between 0 and 1, but its baseline equals the positive class prevalence,

$${AUPRC}_{baseline}= \frac{P}{P+N}$$

where $P$ and $N$ are the numbers of positive and negative samples in the test set. Because AUPRC focuses on precision and recall of the positive class, it is particularly informative under class-imbalanced conditions such as GRN edge prediction.

**Model Implementation Details**

AutoGERN is implemented in Python using PyTorch and PyTorch Geometric. For all experiments, we use a two-layer GNN backbone, with each layer producing 256-dimensional node embeddings. Each layer consists of a message-passing operator selected from the intra-layer search space, followed by Layer Normalization and a nonlinearity. The activation function is also searched over two candidates, ReLU and PReLU.

Architecture search is performed over the joint operation space $\Omega$ using a stochastic, differentiable scheme. For each searchable edge in the supernet, candidate operators are parameterized by a categorical distribution and sampled via a Gumbel–Softmax / Concrete reparameterization, so that in each forward pass only a single operator per edge is active. Network weights $\omega$ are updated on the training split, while architecture parameters $\beta$ are updated using the validation loss. We run 20 epochs of this adaptive architecture search and then fix the discrete architecture by taking the argmax operator per edge. The selected architecture is subsequently fine-tuned for another 20 epochs with the same training protocol.

All models are trained with the Adam optimizer (learning rate 0.0001) and a binary cross-entropy loss on TF–gene pairs. We use a mini-batch size of 128, L2 weight decay of 0.01, and dropout in the GNN and MLP layers with the rate chosen from {0,0.2} based on validation performance. All linear and convolutional layers are initialized with zero-mean normal weights and zero biases. Gradient clipping with a fixed maximum norm is applied at each update step to stabilize training, and a global random seed is used to ensure reproducibility of initialization, architecture sampling, and negative sampling. All experiments (search and final training) are conducted on a single NVIDIA RTX 3090 GPU, on which each dataset comfortably fits in memory and completes within practical runtimes.

**End-to-end usage scenario**

Given a new scRNA-seq dataset, a typical workflow for applying AutoGERN consists of the following steps.

1. Preprocessing workflow includes filtering low-quality cells and lowly expressed genes, normalization of library size, and log-transformation for the counts. We recommend using Scanpy for preprocessing workflow of scRNA-seq data.
2. Then select the target cell type or population of interest and construct a cell-type–specific expression matrix.
3. Define transcription factors and candidate edges: compile a list of TFs using standard TF annotations for the corresponding species and generate candidate TF–gene pairs between these TFs and all expressed genes.
4. Collect prior regulatory evidence for these TF–gene pairs from ChIP-seq or curated regulatory resources (e.g. ENCODE, or cell-type–specific ChIP-based databases) and use it to define positive edges and unlabeled candidate edges of the prior graph.
5. Construct a gene–gene graph where nodes correspond to genes and node features are derived from the cell-type–specific scRNA-seq matrix, and use the TF–gene labels to form supervised training, validation, and test sets.
6. Select a configuration file for the desired model and dataset size (e.g. 500-gene or 1,000-gene setting) and run the provided training script, which automatically performs the stochastic architecture search and retraining stages.

The output consists of a trained AutoGERN model and a ranked list of TF–gene edges with predicted probabilities, which can be thresholded to obtain a GRN or further analyzed for regulatory modules. The accompanying code repository documents the available preprocessing, graph-construction, architecture-search, and evaluation scripts, enabling users to reproduce our experiments or adapt AutoGERN to new scRNA-seq datasets.

**AutoGERN scales with the number of genes and edges**

In Supplementary Figure S3, we present the scalability of AutoGERN in terms of running time and memory usage in two scenarios across seven datasets. On these 14 benchmark datasets, the running time of AutoGERN is highly correlated with the number of edges, showing a linear or even slightly super-linear growth trend. In contrast, the increase in the number of genes has a less significant impact on the time. Additionally, the memory usage of AutoGERN is more sensitive to the growth of data size, with a relatively high cost. It shows a super-linear growth trend as the data size, especially the number of edges, increases. This indicates that the main computational load of the algorithm is determined mainly by the number of inferred edges or the density of the graph structure.

In summary, the performance of AutoGERN gains largely stem from explicit modeling of gene–gene relationships at the link level, which introduces additional computational and memory overhead and thus reduces temporal and spatial efficiency. Further algorithmic optimization and model compression will be important directions for future work.

**Interpretability via illustrative regulatory modules of mDC+500 gene dataset**

We performed an interpretability analysis under the Hard Split setting (which reflects the realistic regime where candidate negatives vastly outnumber positives). We focus this analysis on the mDC-500 dataset because it presents the most severe class imbalance among our benchmarks, making it the most challenging and practically relevant setting to demonstrate that AutoGERN can still recover coherent regulatory modules under extreme imbalance.

Specifically, for mDC-500 we extract a TF-centered regulatory module by selecting the TF hub with the highest out-degree among top-scoring predicted edges and visualizing its top-50 predicted targets, with edge thickness proportional to the predicted score (Supplementary Fig. S4). To assess coherence and biological relevance, we perform GO BP and KEGG enrichment on the top-50 target set (Supplementary Table S2). The module shows significant enrichment for immune-relevant functions, including cytokine-mediated signaling (GO:0019221; $FDR=5.63\times{10}^{-7}$ ), and KEGG pathways such as Th17 cell differentiation ($FDR=1.06\times{10}^{-2}$) and proteasome ($FDR=1.06\times{10}^{-2}$), as well as supportive signals for lysosome-related processes (KEGG “Lysosome”, $FDR=7.52\times{10}^{-2}$). Notably, 13 of the top-50 predicted targets are supported by the ChIP-seq reference network (CST3, CTSH, ATP5G2, HIF1A, AA467197, RAB32, CEBPA, SGK1, BCL1A1A, ILF3, ARL8B, UBE2L6, PSMA1), indicating that the high-confidence module is not only functionally coherent but also partially validated by the reference labels.

## **Supplementary Figures**


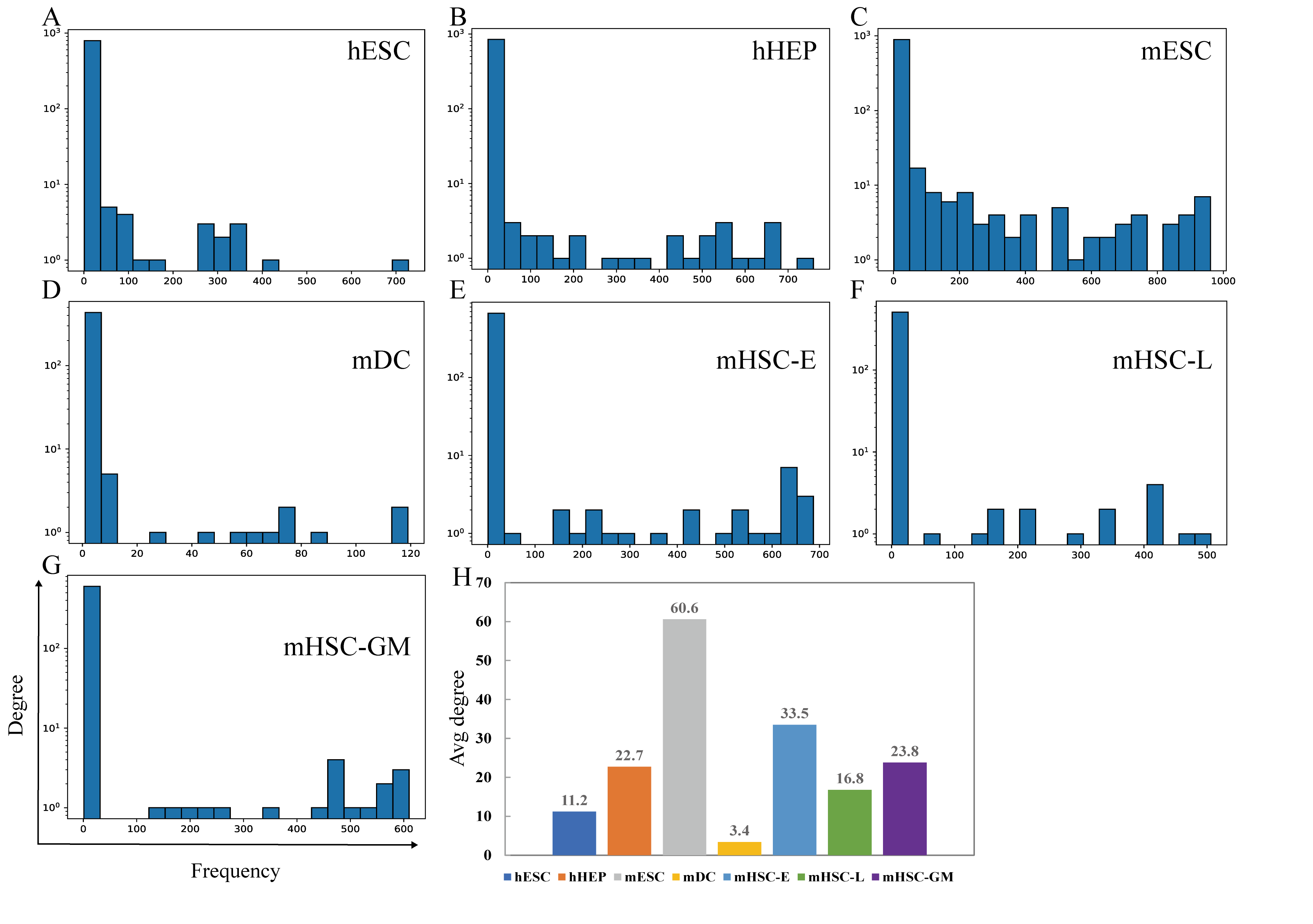


**Figure S1.** **Distribution of nodes in the gold-standard regulatory networks specific to seven scRNA-seq data.** (A-G) show the node distribution of the gold-standard regulatory networks for the seven cell types: hESC, hEHP, mESC, mDC, mHSC-E, mHSC-L, and mHSC-GM, with the vertical axis representing node degree and the horizontal axis representing frequency. (H) The average number of nodes in the gold-standard GRNs for the seven cell types.


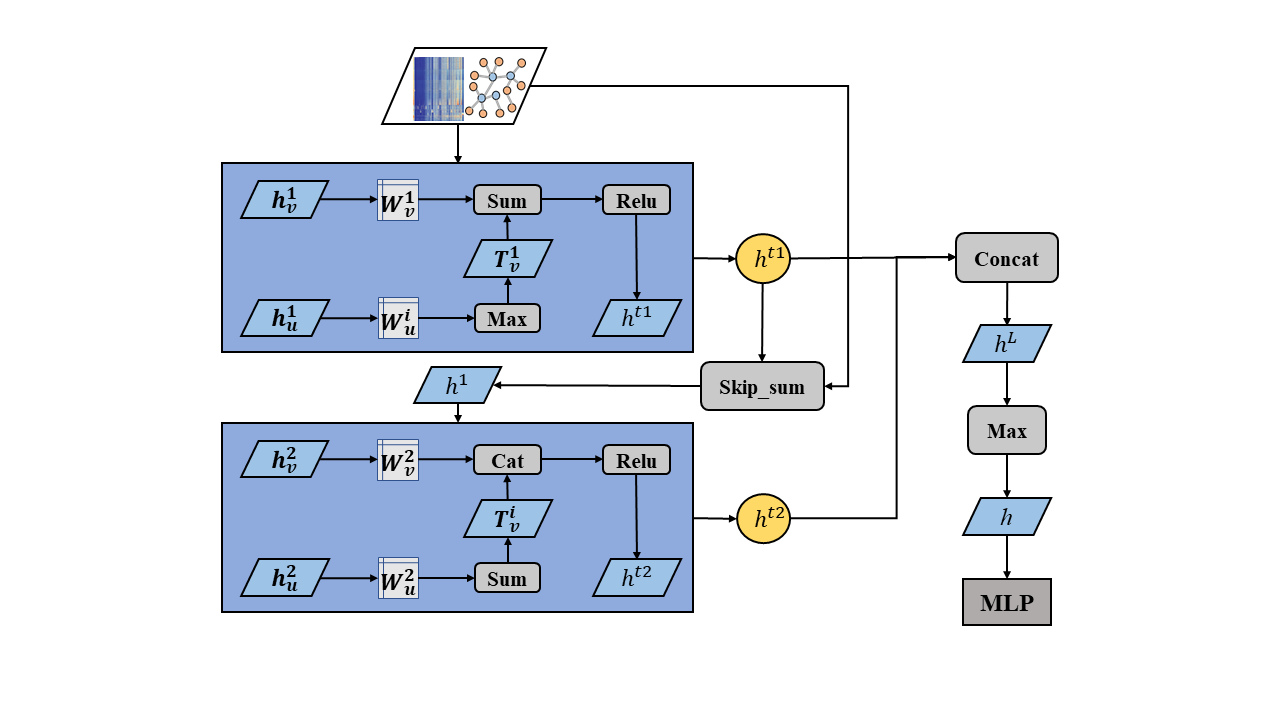


**Figure S2. Visualization of the searched architecture for the hHEP dataset.**

**
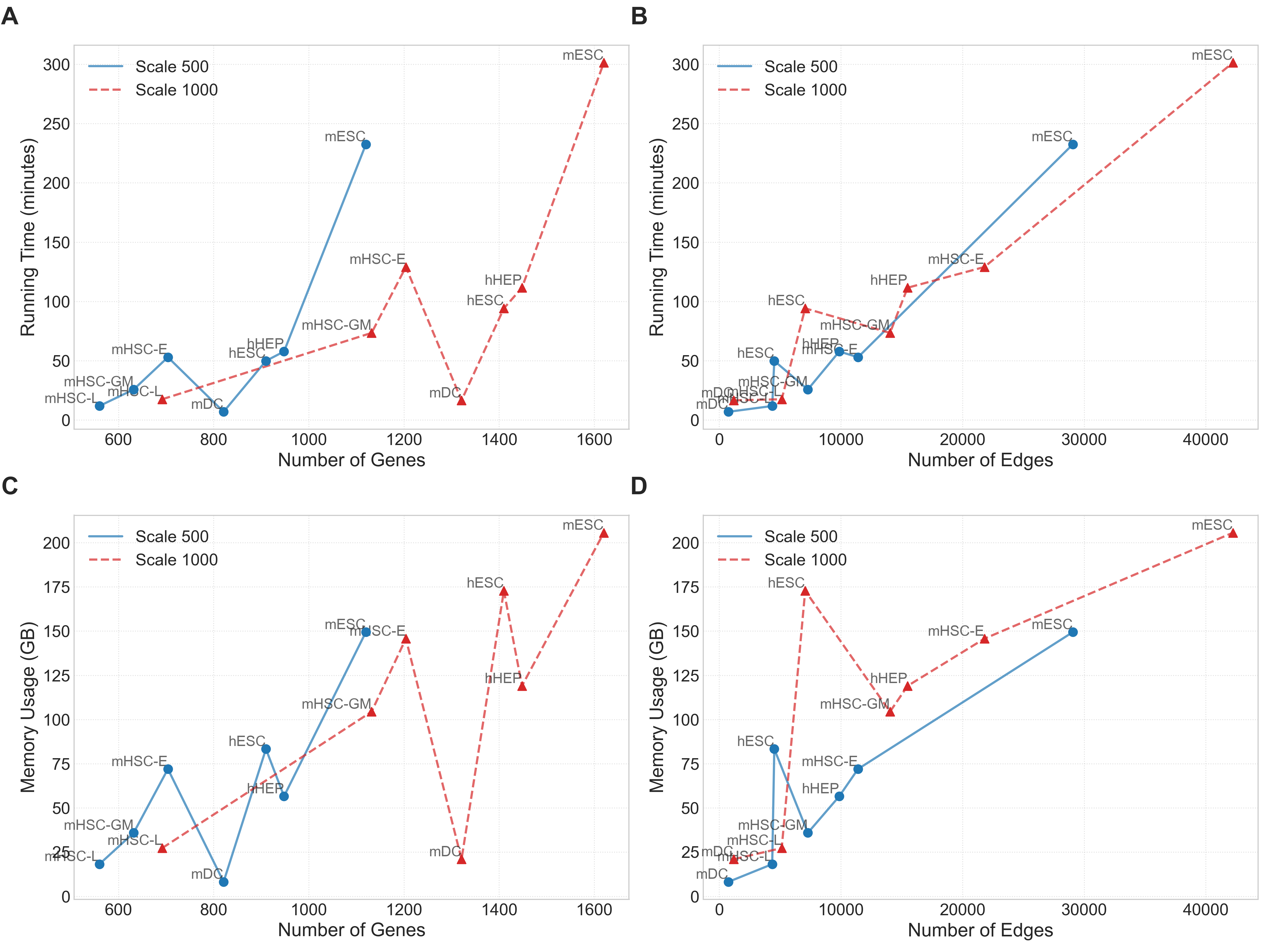
**

**Figure S3. Computational complexity and efficiency of AutoGERN on seven single-cell RNA sequencing datasets.** The trend of running time as the number of genes (A) and the number of edges (B) increase; The trend of memory usage as the number of genes (C) and the number of edges (D) increase.

**
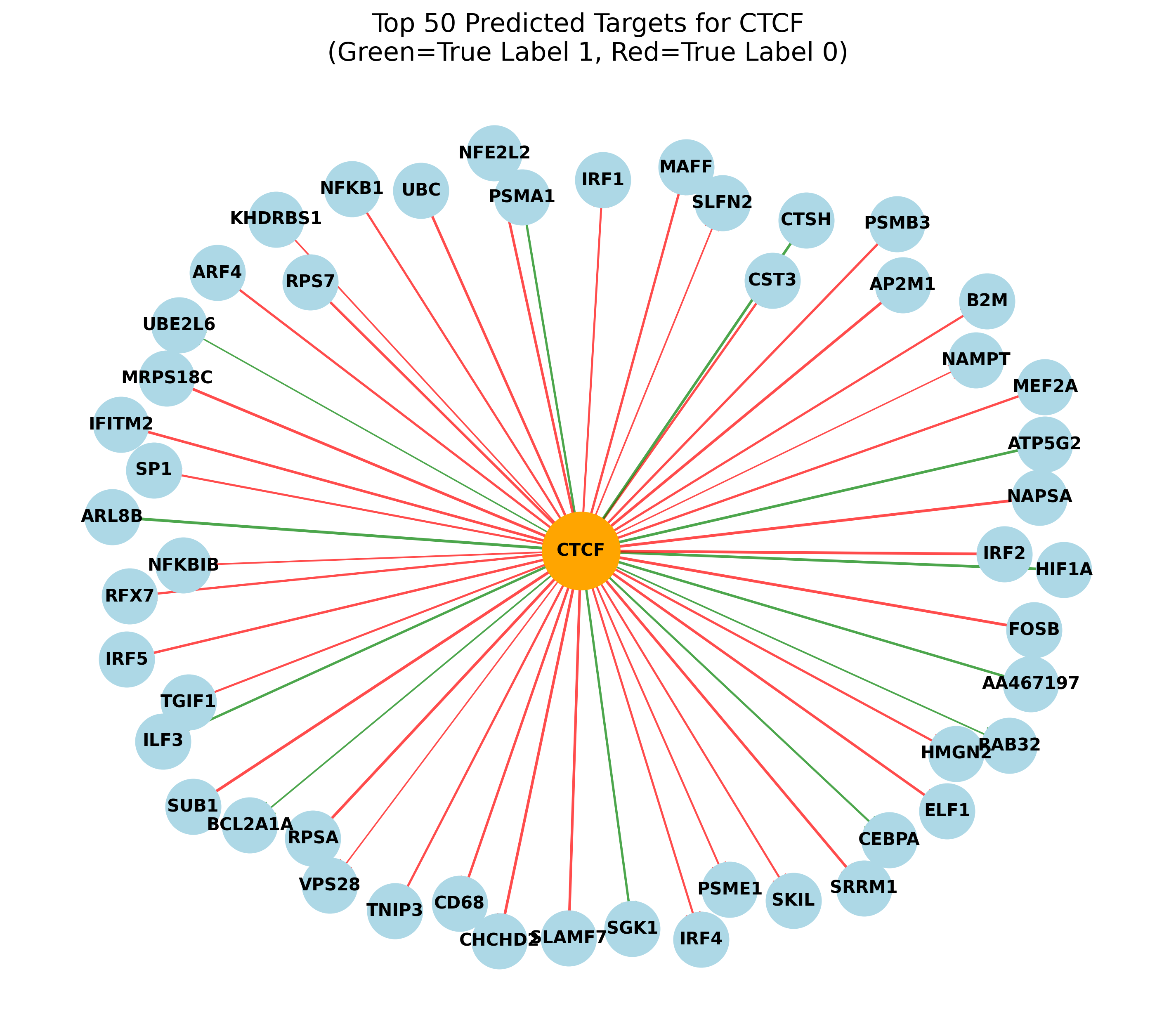
**

**Figure S4. Top-50 targets of CTCF in the inferred GRNs of mDC+500 genes dataset.** The width of edges in the network is proportional to their predicted probabilities.

## **Supplementary Tables**

**Table S1. Quantitative summary of seven single-cell RNA sequencing datasets.** For columns “# TF–gene pairs” and “Edge density”, values are reported as “500-gene / 1000-gene”.

| **Dataset** | **# of cells** | **# of genes** | **# of TFs** | **# of TF-gene pairs** | **Network density** |
| --- | --- | --- | --- | --- | --- |
| hESC | 758 | 11735 | 34 | 4526/7084 | 0.167/0.165 |
| hHEP | 425 | 11515 | 27 | 9939/15556 | 0.379/0.377 |
| mDC | 421 | 7371 | 20 | 756/1193 | 0.085/0.082 |
| mESC | 383 | 18385 | 77 | 21315/42795 | 0.345/0.347 |
| mHSC-E | 1071 | 4762 | 29 | 11557/21975 | 0.578/0.566 |
| mHSC-GM | 847 | 4762 | 22 | 7364/14135 | 0.543/0.565 |
| mHSC-L | 889 | 4762 | 16 | 4398/5180 | 0.525/0.507 |

**Table S2. Top-5 GO (BP) and KEGG enrichment terms of the top-50 targets of CTCF in inferred GRNs of mDC+500 genes dataset.**

| **Genes** | **Term** | **P-value** | **Adjusted P-value** |
| --- | --- | --- | --- |
| **GO (BP)** | | | |
| MEF2A;ARF4;CEBPA;HIF1A;NFKB1;ELF1;CHCHD2;SP1;IRF4;IRF1;TNIP3;SUB1;IRF2;MAFF;NAMPT;UBC;IRF5;NFE2L2 | positive regulation of transcription by RNA polymerase II (GO:0045944) | 2.598377e-12 | 1.829257e-09 |
| MEF2A;ARF4;CEBPA;HIF1A;NFKB1;ILF3;ELF1;CHCHD2;SP1;IRF4;IRF1;TNIP3;IRF2;MAFF;NAMPT;UBC;IRF5;NFE2L2 | positive regulation of transcription, DNA-templated (GO:0045893) | 2.043906e-10 | 7.194551e-08 |
| CEBPA;IFITM2;HIF1A;NFKB1;PSMA1;IRF4;PSMB3;IRF1;IRF2;UBC;PSME1;IRF5;B2M | cytokine-mediated signaling pathway (GO:0019221) | 2.716662e-09 | 5.626814e-07 |
| TGIF1;MEF2A;ARF4;CEBPA;HIF1A;NFKB1;ELF1;CHCHD2;SP1;IRF4;RFX7;IRF1;TNIP3;SUB1;IRF2;MAFF;NAMPT;UBC;FOSB;IRF5;SKIL;NFE2L2 | regulation of transcription by RNA polymerase II (GO:0006357) | 3.197053e-09 | 5.626814e-07 |
| PSMA1;PSMB3;UBC;PSME1;HIF1A;NFE2L2 | regulation of transcription from RNA polymerase II promoter in response to hypoxia (GO:0061418) | 3.161878e-08 | 4.451924e-06 |
| **KEGG** | | | |
| IRF4;HIF1A;NFKB1;NFKBIB | Th17 cell differentiation | 0.000123 | 0.010552 |
| PSMA1;PSMB3;PSME1 | Proteasome | 0.000207 | 0.010552 |
| CEBPA;BCL2A1A;NFKB1 | Acute myeloid leukemia | 0.000686 | 0.023318 |
| CEBPA;SP1;BCL2A1A;NFKB1 | Transcriptional misregulation in cancer | 0.001125 | 0.028681 |
| NAPSA;CTSH;CD68 | Lysosome | 0.003687 | 0.075210 |

**Table S3. Impact of negative sampling strategies.**

| Data setting | Negative sampling schema | AUROC | AUPRC |
| --- | --- | --- | --- |
| mDC500 | Uniform random | 0.989 | 0.989 |
| mDC500 | Co-expression-filtered + uniform random | 0.985 | 0.984 |
| mDC500 (HardSplit) | NA | 0.81 | 0.15 |
| mDC500 (HardSplit) | Co-expression-filtered | 0.817 | 0.177 |
